# Torque depression following active shortening is associated with a modulation of cortical and spinal excitation: a history‐dependent study

**DOI:** 10.14814/phy2.13367

**Published:** 2017-08-14

**Authors:** Jordan Grant, Chris J. McNeil, Leah R. Bent, Geoffrey A. Power

**Affiliations:** ^1^ Department of Human Health and Nutritional Sciences College of Biological Sciences University of Guelph Guelph Ontario Canada; ^2^ Centre for Heart, Lung and Vascular Health School of Health and Exercise Sciences University of British Columbia Kelowna British Columbia Canada

**Keywords:** Cervicomedullary motor evoked potential, cervicomedullary stimulation, concentric, electromyographic signal, force depression, motor evoked potential, transcranial magnetic stimulation

## Abstract

The reduction in steady‐state isometric torque following a shortening muscle action when compared to a purely isometric contraction at the same muscle length and level of activation is termed torque depression (TD). The purpose of this study was to investigate spinal and supraspinal neural responses during the TD state of a maximal voluntary activation of the ankle dorsiflexors. Thirteen subjects (10 male) were recruited for the study. To explore alterations in corticospinal excitability during voluntary muscle activation in the TD state, motor evoked potentials (MEPs), cervicomedullary motor evoked potentials (CMEPs), and maximal compound muscle action potentials (Mmax) were elicited during the isometric steady‐state following active shortening (i.e., TD) and the purely isometric condition. A 15% reduction in steady‐state isometric torque (*P* < 0.05) was observed following isokinetic shortening at 40°/sec. Although mean evoked responses (MEP and CMEP) were not different in the TD state as compared with purely isometric state, the changes in evoked responses were inversely related to one another depending on the level of TD. These findings indicate that supraspinal and spinal responses are interrelated in the TD state. Furthermore, antagonist muscle coactivation during the isometric reference contraction was positively related to TD. These findings suggest the possibility of a relationship between the central nervous system and TD in humans. Further work should be performed to definitively link TD to specific spinal interneurons.

## Introduction

Steady‐state isometric torque following a shortening muscle action is less than that produced during a purely isometric contraction at the same muscle length and level of activation (Abbott and Aubert 1952; Edman 1980; Tilp et al. 2009). This shortening‐induced torque depression (TD) is an intrinsic property of skeletal muscle which occurs across muscle fiber types (Joumaa et al. 2015), in animals and humans (Abbott and Aubert 1952; Lee et al. 1999), and during maximal and submaximal electrically‐stimulated and voluntary contractions (Rousanoglou et al. 2007; Ruiter et al. 1998; Power et al. 2014). The magnitude of the force (Abbott and Aubert 1952; Edman 1975; Herzog and Leonard 1997) and displacement (Abbott and Aubert 1952; Maréchal and Plaghki 1979; De Ruiter and De Haan 2003) during muscle shortening are directly related to the level of TD. Thus, as the work of shortening performed by the agonist muscle increases, the magnitude of the TD becomes greater (Granzier and Pollack 1989; Herzog et al., 2000; Ruiter et al. 1998; Kosterina et al. 2008). Though the phenomenon of TD is well documented across all structural levels of muscle, very little is known regarding the effect TD may have on the central nervous system. The potential for TD to induce central neural adaptations therefore requires further investigation as it may influence the voluntary control of movement.

The long‐lasting nature of TD following muscle shortening (20–30 s) has been linked to a reduction in the total number of force producing actin‐myosin cross‐bridges in the new thick and thin filament overlap zone (Herzog et al. 1998). Evidence in support of this relationship has been provided with the observation of decreased whole muscle (Herzog et al. 1998), muscle fiber (Joumaa et al. 2015) and myofibril (Joumaa and Herzog 2010) stiffness, along with the abolishment of TD after a brief period of muscle relaxation (Abbott and Aubert 1952; Herzog et al. 1998). A stress‐induced angular deformation of the thin actin filament in both the new (Maréchal and Plaghki 1979; Daniel et al. 1998) and previous actin‐myosin overlap zones, as well as a reduction in the average force per cross‐bridge (Joumaa et al. 2012) are generally accepted as the mechanical foundations of TD.

During movement at the whole organism level, given that TD is a measure of a state‐dependent reduction in the net torque about a joint, it is important to consider the action of the agonist and antagonist muscles during voluntary contractions. Tilp et al. (2009) found an 18% reduction in torque following active shortening of the dorsiflexors and no change in the tibialis anterior (TA) electromyographic signal (EMG). During maximal contractions, most previous research concurs with these findings, displaying no change in agonist activation between the TD and isometric reference (ISO) contractions (Lee et al. 1999; Lee and Herzog 2003). However, recent findings (Jones et al. 2016) have shown lower EMG amplitude in the TD state during maximal voluntary contractions (MVCs), with no change in EMG median power frequency or voluntary activation. During submaximal contractions (30% MVC) of the adductor pollicis, Rousanoglou et al. (2007) found an 18% increase in average full wave rectified EMG when matching ISO force in the TD state. This elevation in EMG was attributed to a compensatory increase in muscle activation employed to match ISO levels of force. With respect to antagonist muscle activation, there is a large body of evidence which shows no change following muscle shortening so it is presumed not to influence TD (De Ruiter and De Haan 2003; Lee and Herzog 2003; Rousanoglou et al. 2007; Tilp et al. 2009; McGowan et al. 2010; Kosterina et al. 2013; Power et al. 2014; Jones et al. 2016).

Surface EMG is a measure of central and peripheral neural output and may therefore become modified in a number of ways. The key aspect of shortening‐induced TD is the reduction in muscle force during a state of maximal neuromuscular activation. It is therefore possible that this discrepancy in the EMG‐force reationship might influence neuromotor feedback and alter neuromuscular activation strategies. The Golgi Tendon Organ (GTO), a peripheral force transducer involved in the sensation of force at the muscle, excites the Ib‐inhibitory interneuron within the spinal cord which inhibits motoneurone (MN) discharge (Binder et al. 1977; Crago et al. 1982). Another potential site of spinal modulation, the Renshaw cell, is activated by the firing of the MN and provides negative feedback to gamma MNs (Ellaway 1971) and the alpha MN (Eccles et al. 1954). In addition to their effect on the MN pool of the agonist muscle, similar to Ib‐inhibition, Renshaw cells also indirectly excite the MN pool of the antagonists via negative feedback to Ia‐inhibitory interneurons (Hultborn et al. 1971). Though muscle activation (as measured with surface EMG) appears to be mostly unaffected by TD, it is not known if the excitability of the central nervous system, specifically the MN pool, within the spinal cord (spinal excitability), or the motor cortex (supraspinal excitability) is altered in the steady‐state following active muscle shortening.

The aim of this study is to determine spinal and supraspinal excitability during maximal ISO and TD states. Owing to the findings of previous studies which have shown no change in EMG between TD and ISO states during MVC (Abbott and Aubert 1952; Tilp et al. 2009), we hypothesize that any change at one level of the central nervous system (i.e., spinal or supraspinal) will be counterbalanced by an opposite change at the other level of the central nervous system. Such findings would indicate that the shortening‐induced disruption of the isometric EMG‐force relationship alters the voluntary control of force through spinal and cortical neural pathways.

## Materials and Methods

### Ethical approval

All participants gave written informed consent and all procedures were approved by the Research Ethics Board of the University of Guelph and conformed to the Declaration of Helsinki.

### Participants

Thirteen healthy participants with a mean age of 23 ± 1 year (10 males), height of 179 ± 3 cm and weight of 75 ± 3 kg were recruited for participation in the study. Participant data were collected within a single session. With respect to TD, nonresponders are identified as participants with negative TD on average (i.e., an increase in torque after shortening). One nonresponder was identified and removed from all analyses (i.e., torque, EMG, evoked responses). Therefore, data from 12 participants were used for analysis.

### Experimental set‐up

A HUMAC NORM dynamometer (CSMi Medial Solutions, Stoughton, MA) was used for all torque, angular velocity and position recordings. The participants were seated with their right hip at 110° of flexion and right knee at 140° of flexion. The right knee was immobilized, with the dynamometer's leg restraint positioned superior and a cushion placed inferior. Movement at the torso was restricted with a four‐point seatbelt harness. The right foot was fixed to a dorsi/plantar flexor dynamometer pedal‐adaptor with one inelastic strap placed over the ankle and another at the mid‐distal portion of the metatarsals. The maximum ankle dorsiflexion and plantar flexion (PF) angles were set to 100° and 140° plantar flexion, respectively, allowing for 40° of ankle excursion.

Prior to EMG electrode placement, skin locations were thoroughly cleaned with alcohol swabs. Silver‐silver chloride (Ag/AgCl) electrodes (1.5 × 1 cm: Kendall, Mansfield, MA) were used for all recordings in a monopolar configuration to optimize the acquisition of evoked potentials. One electrode was positioned over the tibialis anterior (TA) approximately 7 cm inferior and 2 cm lateral to the tibial tuberosity, a second electrode was placed over the distal tendon of the tibialis anterior, and a ground electrode was placed on the patella. To record antagonist muscle activation, one electrode was placed on the soleus along the midline of the leg approximately 2 cm inferior to the border of the gastrocnemius, and a second electrode was placed on the calcaneal tendon.

Isometric torque, concentric torque, angular position and stimulus trigger data were sampled at 1000 Hz using a 12‐bit analog‐to‐digital converter (PowerLab System 16/35, ADInstruments, Bella Vista, Australia). EMG data were sampled at 2000 Hz, and band pass filtered (10 Hz–1000 Hz). All data were analyzed with Labchart (Labchart, Pro Modules 2014, version 8) software.

### Deep fibular and tibial nerve stimulation

To normalize voluntary and evoked EMG, maximal compound muscle action potentials (Mmax) were recorded at the tibialis anterior and soleus muscles by stimulating the deep fibular and tibial nerves, respectively, with a standard clinical bar electrode (Empi, St Paul, Minnesota, USA), coated in conductive gel. The deep fibular nerve was located by palpating the head of the fibula and moving in a distal and posterior direction, until the nerve was intercepted. The tibial nerve, innervating the plantar flexor muscles, was found by locating the distal tendon of the semitendinosus muscle and moving laterally while palpating deep into the popliteal fossa. All peripheral nerve stimuli were delivered as single pulses from a constant current high voltage stimulator (model DS7AH, Digitimer, Welwyn Garden City, Hertfordshire, UK). Pulse width was set to 200 *μ*s and voltage was continuously variable between 100 and 400V. With the participant relaxed, current was incrementally elevated, starting from 20 mA, until the amplitude of the resting M‐wave reached a plateau (Mmax). To ensure the consistent activation of all motor units, the current was adjusted to a supramaximal level, equivalent to 110% of that required to generate Mmax.

### MVC and voluntary activation

Participants were verbally encouraged during all MVCs and the torque trace was visible throughout all experimental trials (Gandevia 2001). The interpolated twitch technique was used to evaluate voluntary activation during a single MVC, prior to experimental MVCs (Belanger and McComas 1981; Power et al. 2014). The peak torque resulting from an interpolated twitch, presented during the plateau phase of the MVC, was compared to a resting twitch delivered 1–2 sec after the MVC. All subjects were capable of reaching a minimum of 95% VA. The level of activation (VA) was calculated as:

$$\begin{array}{cc} \text{VA}\% & =[1-(\text{interpolated twitch torque/resting twitch}\\ & \text{torque})]\times 100 \end{array}$$

### Cervicomedullary stimulation

Two Ag/AgCl electrodes (10 mm diameter) were used for cervicomedullary stimulation (CMS) to elicit cervicomedullary motor evoked potentials (CMEPs) by passing a current across the corticospinal tract at the level of the mastoids. Electrodes were placed at a location approximately 2 cm medial and superior to the mastoid processes (McNeil et al., 2009). Single pulse stimuli were presented (anode on right side and cathode on left) with the DS7AH at a pulse duration of 200 *μ*s and continuously variable voltage between 100 and 400 V. Current was adjusted (150–300 mV) in order to produce a CMEP with an amplitude equivalent to 40% of resting Mmax while the subject performed a brief MVC.

### Transcranial magnetic stimulation

Motor evoked potentials (MEPs) were elicited with transcranial magnetic stimulation (TMS) of the motor cortex by placing the stimulating coil over the vertex of the skull and slightly to the left (to preferentially activate the dorsiflexors of the right leg). In order to determine an appropriate stimulus intensity, single magnetic pulses were delivered with a double cone coil (110 mm) linked to two Magstim 200^2^ stimulators (Magstim, Dyfed, UK) while the subject performed a brief MVC. Stimulus intensity (25–90% stimulator output) was adjusted until an MEP amplitude corresponding to 40% of Mmax was recorded from the TA.

### Experimental procedures

**Figure 1:**
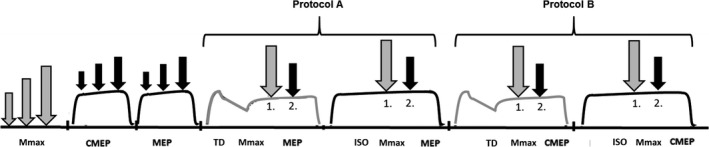
Experimental timeline. Deep fibular nerve stimulation (gray arrow) was delivered at rest to determine the maximal M‐wave (Mmax). Cervicomedullary stimulation or transcranial magnetic stimulation (black arrow) was delivered during brief (~2 sec) MVCs to set CMEP and MEP amplitudes to ~40% Mmax. During 7 sec MVCs, M‐waves and MEPs were collected in a torque depressed (TD) contraction and an isometric control (ISO) contraction (protocol A). Then, M‐waves and CMEPs were collected in TD and ISO states during 7 sec MVCs (protocol B). Protocols A and B were performed three times. As described in the text, time points 1 and 2 indicate the timing of deep fibular nerve stimulation (1) and CMS or TMS (2).

The experimental timeline is shown in Figure 1. Each TD trial was followed by an ISO trial (control trial). Protocol A was followed by protocol B and this sequence was repeated a total of three times. Therefore, three TD trials and three ISO trials were performed for each of the two protocols (TMS and CMS) for a total of 12 MVCs. A 5 min rest period followed all experimental trials.

#### Protocol “A”: assessment of cortical and peripheral excitability

For each TD trial, the protocol consisted of a 1 sec isometric MVC phase at 140° PF, 1 sec isokinetic MVC shortening phase (40°/sec) and 5 sec isometric MVC phase at 100° PF. A stimulus was manually delivered to the deep fibular nerve at the 5th second (time point 1) and the TMS pulse was administered at the 6th second (time point 2) of the 7 sec MVC. Protocol A is outlined in Figure 1. A total of 3 Mmax and MEPs were collected under both TD and ISO conditions for Protocol A.

For the ISO trials, the ankle was set to an angle of 100°. An isometric MVC was performed for 7 s with stimulation of the deep fibular nerve occurring at the 5th second (time point 1) and the TMS pulse at the 6th second (time point 2) of the trial.

#### Protocol “B”: assessment of spinal excitability

MVC parameters were identical to those of Protocol A but the type of stimulation differed. That is, CMS was administered at the 6th second (time point 2) of the TD trial to correspond with the delivery of TMS in Protocol A. Protocol B is outlined in Figure 1. A total of 3 CMEPs were collected under both TD and ISO conditions for Protocol B.

#### Data analysis and statistics

Mean isometric torque and root mean squared EMG (EMG_RMS_) were calculated over the 500 ms window prior to each stimulus. A paired *t* test was performed to compare the torque and EMG data between TD and ISO trials for each stimulus to validate the presence of TD at the time of stimulation. The EMG_RMS_ of TA and soleus was normalized to the respective M‐wave in order to quantify agonist activation and antagonist coactivation (ACA). Concentric torque was calculated as the mean torque during the 1 sec isokinetic MVC phase in the all of the TD protocols. The mean concentric torque for each participant was then normalized to the respective participant's mean ISO torque prior to time point 1.

In order to detect and subsequently remove outliers from the data set, MEPs and CMEPs were normalized to the subject's median evoked response. The mean of these normalized data was calculated across participants and any response which fell more than two standard deviations above or below the normalized mean was rejected. Four CMEPs and five MEPs were deemed outliers and rejected from analysis.

A paired *t* test was performed for each of the variables between the TD and ISO states to identify changes in peripheral, cortical and spinal excitability in the TD state. Regression analysis was then used to determine if the evoked responses were related to the magnitude of TD in the ISO steady‐state. For each regression, the normalized CMEPs and MEPs were represented as a relative change from the ISO to the TD state. Therefore, CMEPs (% Mmax) were further normalized using the following formula:

$${\text{CMEP}}_{\mathrm{diff}}={\text{CMEP}}_{\mathrm{TD}}/{\text{CMEP}}_{\mathrm{ISO}}-1$$

To account for the influence of changes in spinal excitation on MEPs, MEPs were further normalized to CMEPs with the following formula:

$${\text{MEP}}_{\mathrm{diff}}=\left({\text{MEP}}_{\mathrm{TD}}/{\text{CMEP}}_{\mathrm{TD}}\right)/\left({\text{MEP}}_{\mathrm{ISO}}/{\text{CMEP}}_{\mathrm{ISO}}\right)-1$$

Significance was determined based on a *P*‐value of <0.05. Descriptive data found in text are reported as means ± standard deviation, while that found in figures are reported as means ± standard error of the mean.

## Results

### Torque depression and agonist activation

**Figure 2:**
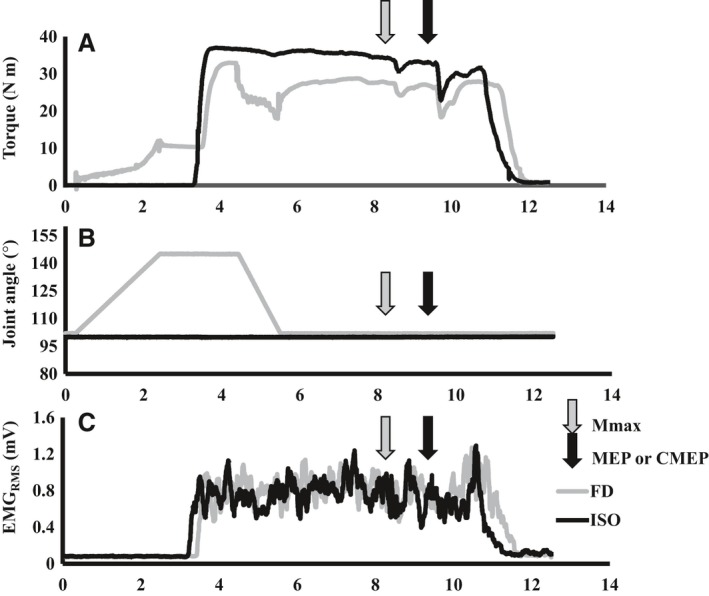
Torque (A), joint angle (B) and agonist EMG (C; moving 500 msec window) during 7 sec MVCs in the torque depressed (TD) and isometric reference (ISO) states for a representative subject. During TD trials (gray traces), an MVC was initiated for 1‐sec at 140° PF. The dynamometer arm then rotated the foot at 40°/sec to an angle of 100° PF and the participant continued to contract for another 5 sec. A supramaximal stimulus was delivered to the deep fibular nerve at time point 1 of the MVC (to elicit an Mmax) and a TMS or CMS pulse was administered at time point 2 (to elicit a MEP or CMEP, respectively). During ISO trials (black traces), the same stimulus timing was used as in the TD trial, only the ankle was fixed at an angle of 100° PF.

**Figure 3:**
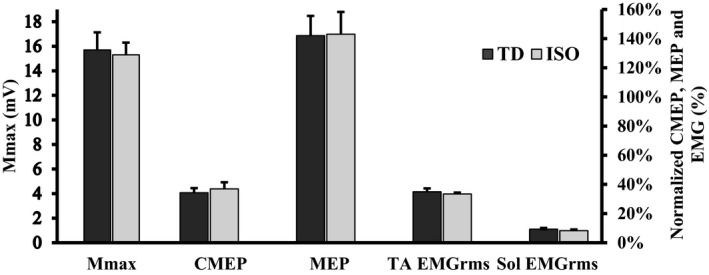
Absolute peak‐to‐peak amplitude of Mmax as well as normalized values of CMEP amplitude (% Mmax), MEP amplitude (% CMEP) and EMG_RMS_ of the TA and soleus (% Mmax) in the TD and isometric reference contractions. Columns are group means ± SEM. None of the variables differed between ISO and TD states (*P* > 0.05).

ISO‐control torque did not change between protocol A or protocol B (*A* = 27.0 N·m ± 7.2 N·m, *B* = 23.0 N·m ± 7.0 N·m, *P* > 0.05), or from the first to last MVC of the study (MVC1: 23.5 N·m ± 7.1 N·m, MVC12: 22.4 N·m ± 6.4 N·m). TD was successfully induced as the steady‐state torque following active shortening was lower as compared with a purely isometric MVC (average between Protocols A and B: TD = 15.6 ± 1.8% and 15.1 ± 2.2% at time points 1 and 2, respectively; *P* < 0.05 – Fig. 2). The level of TD varied across subjects and, when binned in increments of 5%, there was: 1 subject at 0–5%, 2 subjects at 5–10%, 3 subjects at 10–15%, 3 at subjects 15–20%, and 3 subjects at 20–25%. Mean TA‐EMG_RMS_ (% Mmax) was not different between TD and ISO states at time point 1 (TD: 36.0 ± 2.7%; ISO: 33.6 ± 3.1%, *P* > 0.05) or time point 2 (TD: 36.0 ± 2.6%; ISO: 33.6 ± 3.1%, *P* > 0.05 – Fig. 3) indicating no change in mean MN output.

### Antagonist coactivation

ACA was not different for the TD (9.3 ± 1.0%) and ISO (8.4 ± 1.0%) conditions at time point 1 (*P* > 0.05). Similarly, the antagonist muscles were equally active in the TD (9.3 ± 0.9%) and ISO (8.3 ± 0.8%) at time point 2 (*P* > 0.05).

**Figure 4:**
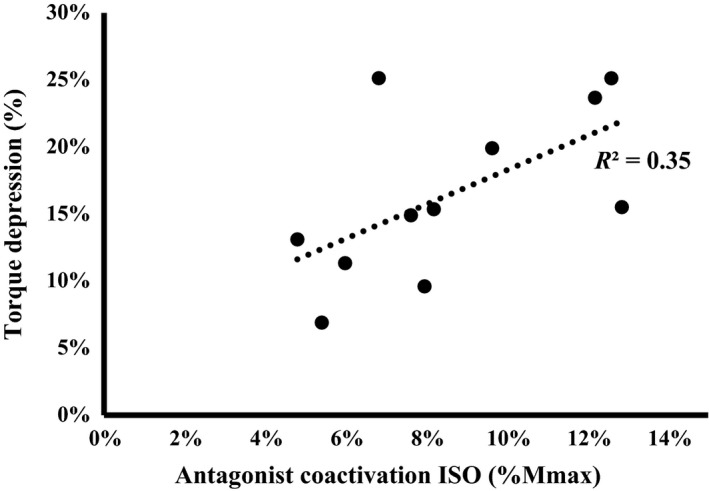
The relationship between antagonist muscle (soleus) coactivation (ACA) during the isometric reference contraction and the magnitude of torque depression (TD). Each data point represents the mean values for an individual participant. Regression analysis revealed a significant association (*R* ^2^ = 0.35, *P* = 0.024), such that those participants with higher levels of ACA also had greater TD.

**Figure 5:**
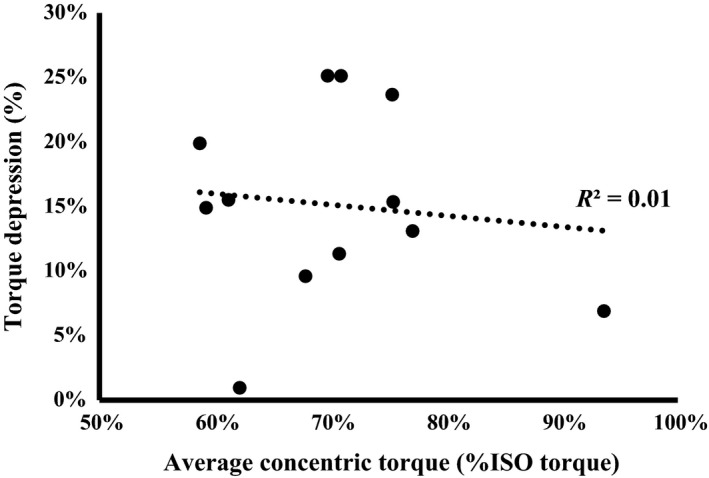
The relationship between mean concentric torque during the dynamic phase of the torque depression (TD) contractions and the magnitude of TD. Each data point represents the mean values for an individual participant. No association was found between the two variables (*R* ^2^ = 0.01, *P* > 0.05).

To assess if a relationship existed between ACA and the magnitude of TD, a regression was performed between ACA in the ISO state and TD magnitude. The regression analysis revealed a strong positive relationship (*R* ^2^ = 0.35, *P* = 0.024) between TD and ACA (Fig. 4). No relationship was found between the magnitude of TD and EMG_RMS_ of the TA (% Mmax) in the ISO state (*R* ^2^ = 0.02, *P* > 0.05) or the magnitude of TD and average concentric torque (*R* ^2^ = 0.01, *P* > 0.05 – Fig. 5).

### Evoked responses

**Figure 6:**
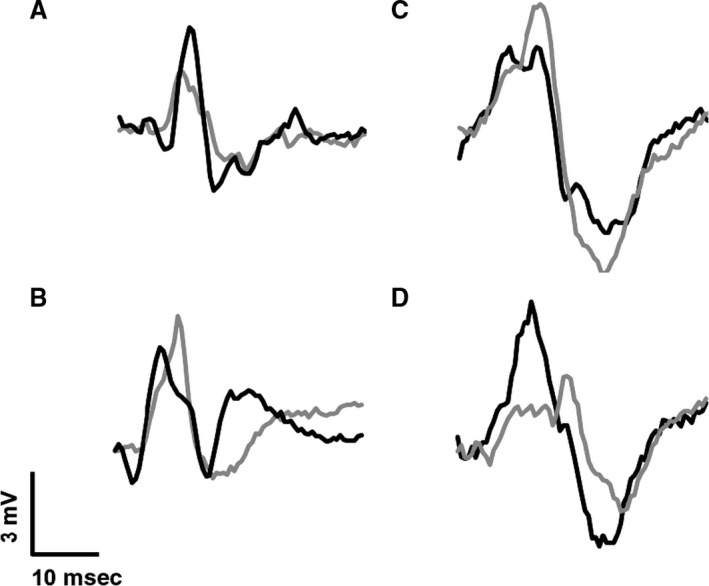
Raw data trace for two participants MEP and CMEP in the isometric condition (black line) and torque depressed steady‐state (gray line). Participant 1 MEP (A) and CMEP (B), participant 2 MEP (C) and CMEP (D).

Mmax, CMEP and MEP peak‐to‐peak amplitude did not change between the TD and ISO states (*P* > 0.05 – raw data trace Fig. 6). Mean normalized CMEP (normalized to Mmax) and MEP (normalized to CMEP) amplitude also remained constant between MVC types (*P* > 0.05 – Fig. 3).

**Figure 7:**
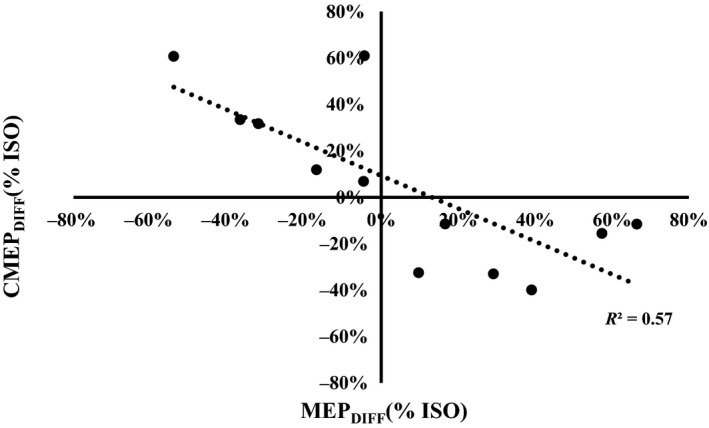
The relationship between the change in MEP amplitude (*x*‐axis) and CMEP amplitude (*y*‐axis) during the torque depressed (TD) state. A significant negative relationship was found between the change in MEP and change in CMEP (*R* ^2^ = 0.57, *P* < 0.05).

The standard analysis technique used to interpret spinal and cortical excitation with CMS and TMS holds the assumption that the independent variables are binomial in nature. Some examples in which the binary approach has been used include comparisons of means between fatigued and nonfatigued states (McNeil et al. 2011), force enhanced and control states (Hahn et al. 2012), and pathological and healthy participants (Ridding et al. 1995). In this study, the average TD was highly variable across participants (range: 2–26%), indicating that the TD state exists over a spectrum and not simply as a binomial, thus implying that a regression may be a more effective analysis strategy. The change in normalized CMEP (normalized to Mmax) amplitude was plotted against the change in normalized MEP (normalized to CMEP). With this approach, negative values for CMEP_diff_ and MEP_diff_ reflect a change toward spinal and cortical inhibition or disfacilitation, and positive CMEP_diff_ and MEP_diff_ values represent a change toward spinal and cortical excitation in the TD state, respectively. The MEP and CMEP in the TD state followed a significant negative relationship (*P* = 0.004, *R* ^2^ = 0.57 – Fig. 7).

## Discussion

This study revealed that normalized mean evoked responses, representing spinal and supraspinal excitability, did not change between TD and ISO states. Spinal and supraspinal excitability were, however, negatively related to one another, confirming our hypothesis that an excitatory or inhibitory change at the spinal level would be counterbalanced by an opposite change at the supraspinal level. Futhermore, this study identifies a positive relationship between the activation of the antagonist muscles during the ISO state and the magnitude of TD.

The average shortening‐induced TD observed in thisstudy was similar to the findings of other experiments utilizing contractions of the dorsiflexors (Tilp et al. 2009; Power et al. 2014), and other muscle groups (Lee et al. 1999; Rousanoglou et al. 2007; Jones et al. 2016). Furthermore, the average level of activation of the agonist and antagonist muscles, as indicated by EMG_RMS_, was not different in the TD and ISO states, which supports much of the literature (Tilp et al. 2009; Power et al. 2014).

### Spinal and supraspinal excitability and EMG in the TD state

The common method of analyzing state‐dependent changes in spinal, supraspinal and peripheral excitability compares the mean CMEP (normalized to Mmax), MEP (normalized to either Mmax or CMEP), and Mmax values, respectively (Martin et al. 2006; McNeil et al. 2011). When this method was performed with the data in the current study, no changes in CMEP, MEP, Mmax or EMG_RMS_ were observed (Fig. 3). Martin et al. (2006) found that spinal excitability follows an inverted “U” relationship with isometric force in the biceps brachii, reaching a peak response at 75% MVC and tapering downward from 75% to 100% MVC. Spinal (motoneurone) excitability is therefore influenced by the level of descending drive (i.e., trajectory of the afterhyperpolarization or recurrent inhibition via Reshaw cells) but we suggest it may also be influenced by the level of force at the muscle (i.e., modulating Ib inhibitory feedback). The range of TD observed across responders in the current study was fairly broad (2–26% TD). Based on this range of TD values, it was thought that some participants may have varying inhibitory feedback, given the variability in force acting at the muscle. For this reason, CMEP and EMG_RMS_ were normalized to Mmax (in order to control for the possibility of a change in peripheral excitability) and scored as a percent change from the ISO to the TD state. Furthermore, the MEP was normalized to CMEP in order to account for changes in spinal and peripheral excitability which may influence MEP amplitude. When MEPs were plotted against CMEPs, a significant negative relationship was observed (Fig. 7). That is, state‐dependent transitions toward spinal excitation were accompanied by reductions in supraspinal excitation, whereas shifts toward spinal inhibition/disfacilitation were accompanied by greater supraspinal excitation. Therefore, the binary statistical method used (t‐test) failed to link alterations in spinal and supraspinal excitability to the TD state as a result of the bidirectional transitions of these responses in the TD state, effectively balancing the group means to infer no change.

Though our data set does not allow us to identify specific interactions at the spinal and supraspinal levels or any possible influence on the magnitude of TD, based on the literature, we will speculate on the role of Ia and Ib afferents. The rise in spinal excitation in the TD state for some participants may be attributed to a reduction in GTO firing, owing to less tension at the muscle (Jami 1992), and may, in fact be linked to the reduction in supraspinal excitability to prevent supramaximal excitation of the MN. Perhaps the more excitable MN in the TD state causes an increase in Renhaw cell discharge which could, in turn, decrease Ia input to the cortex and potentially decrease cortical drive. Evidence of Ia afferent projections to the cortex in humans have been shown for lower (Starr et al. 1981; Macefield et al. 1989) and upper limb (Gandevia and Burke 1990) muscles. For those participants who displayed a shift toward spinal inhibition/disfacilitation and higher supraspinal excitability in the TD state, it is thought that the magnitude of the TD was insufficient to disengage the GTO to the same extent as the participants who displayed reduced spinal excitability. Given that Ib afferents also have supraspinal projections (Landgren and Silfvenius 1969), it is possible that these participants increased cortical drive in an attempt to overcome the TD, accounting for the facilitated supraspinal response. As all contractions were maximal, Renshaw cells likely inhibited additional descending signals to “cap” neuromuscular activation (Eccles et al. 1954), explaining the trend toward spinal inhibition/disfacilitation.

To date, the only other study which has investigated cortical and spinal modulation in the history‐depedent state (i.e., residual force enhancement; RFE) was conducted by Hahn et al. (2012). These authors assessed the MEP and CMEP with RFE, where torque during the isometric steady‐state following active lengthening is greater than the torque attained with a purely isometric contraction at the same muscle length and level of activation. Using similar techniques to those outlined in this experiment, RFE was shown to be accompanied by an increase in MEP area but no change in CMEP area (both potentials normalized to Mmax). Given that the increase in MEP occurred with no change in CMEP, these findings were interpreted as an increase in supraspinal excitability. Though it could not be determined if the change in neural excitation accounted for some of the enhancement of force, the data did demonstrate an altered neural response to the RFE state. This study, extends these history‐dependent neural adaptations to include the phenomenon of TD during MVCs.

### Torque during shortening (concentric) and antagonist coactivation

Increasing the amount of work performed during shortening, either by increasing the displacement or torque, or decreasing the shortening velocity (to increase concentric torque) has been shown to increase TD (Leonard and Herzog 2005). In the present study, displacement and angular velocity were held constant, therefore the only impact on work was due to the between‐subject variation in maximal concentric torque. Participants with higher concentric torque (normalized to their isometric MVC) did not have greater TD than those with lower concentric torque (Fig. 5). Typically, changes in TD are seen with relatively large manipulations of work (Herzog et al. 2000). The variation in concentric torque observed across participants in the present study was minimal (range: 59–94% isometric MVC torque) and may not have been sufficiently broad as to influence TD.

Antagonist coactivation (ACA) has been shown not to change from the ISO to the TD state and has thus been stipulated as a non‐contributer to TD (Rousanoglou et al. 2007; Tilp et al. 2009; Power et al. 2014; Jones et al. 2016). While the present study supports previous findings of no change in average ACA in the TD state (Fig. 3), it provides evidence to suggest that the activation of the antagonist muscles is, in fact, linked to the TD state. TD increased linearly with the participant average ACA during the ISO MVCs (*R* ^2^ = 0.35), indicating that participants with greater activation of the antagonist muscles had greater TD (Fig. 4). Recorded TD reflects a net change in torque about the joint and is therefore affected by the agonist/antagonist torque ratio. Thus, as the activation of the antagonist muscles increases, the net TD about the joint becomes larger, even if the absolute reduction in torque at the agonist muscle is held constant. Furthermore, given the influence of Ib‐inhibitory interneurons and Renshaw cells on the antagonist muscle, the link between TD and antagonist muscle activation could also exist as a consequence of the aformentioned change in spinal and supraspinal excitation. Given that the ACA‐TD relationship was significant and the concentric torque‐TD relationship was not, the role of the antagonist muscles should be taken into consideration when investigating TD with voluntary contractions.

## Conclusion

Evoked responses, when represented as participant mean data in a binary fashion, were not different between the TD and ISO states. When spinal and supraspinal responses were instead represented as a spectrum, TD was linked to neural adaptations which may involve both Renshaw cells and Ib‐inhibitory interneurons. Evidence was also found to suggest that the antagonist muscles are, in fact, related the TD state, identifying another variable which may influence the history‐dependence of torque during voluntary contractions. TD may therefore modify, as well as become modified by neuromuscular activation strategies, suggesting that TD and the voluntary control of movement are bidirectionally linked.

## Conflict of Interest

The authors declare no competing interests.
